# Thyroid Carcinoma Glycoproteins Express Altered *N*-Glycans with 3-O-Sulfated Galactose Residues

**DOI:** 10.3390/biom14121482

**Published:** 2024-11-21

**Authors:** Jordan M. Broekhuis, Dongli Lu, Rajindra P. Aryal, Yasuyuki Matsumoto, Lauren E. Pepi, Natalia Chaves, Jorge L. Gomez-Mayorga, Benjamin C. James, Richard D. Cummings

**Affiliations:** Department of Surgery, Beth Israel Deaconess Medical Center, Harvard Medical School, Boston, MA 02215, USA; jbroekhuis@mgb.org (J.M.B.); dongli.lu@cshs.org (D.L.); rparyal@bidmc.harvard.edu (R.P.A.); yasuyuki.matsumoto@fda.hhs.gov (Y.M.); lpepi@bidmc.harvard.edu (L.E.P.); nchaves1@bidmc.harvard.edu (N.C.); jgomez7@bidmc.harvard.edu (J.L.G.-M.); bjames1@bidmc.harvard.edu (B.C.J.)

**Keywords:** GlcNAc bisection, glycosylation, lectin histochemistry, mass spectrometry, papillary thyroid cancer, RNAseq, sialylation, sulfation, sulfoglycomics, thyroid carcinoma, western blotting

## Abstract

Aberrant protein glycosylation is a hallmark alteration of cancer and is highly associated with cancer progression. Papillary thyroid cancer (PTC) is the most common type of thyroid cancer, but the *N*-glycosylation of its glycoproteins has not been well characterized. In this work, we analyzed multiple freshly prepared PTC specimens along with paired normal tissue obtained from thyroidectomies. Glycomic analyses focused on Asn-linked (*N*)-glycans and employed mass spectrometry (MS), along with Western blot approaches of total solubilized materials that were examined for binding by specific lectins and a monoclonal antibody (mAb) O6, specific for 3-O-sulfated galactose residues. We observed major differences in PTC versus paired normal specimens, as PTC specimens exhibited higher levels of *N*-glycan branching and bisection with *N*-acetylglucosamine residues, consistent with RNAseq data. We also found that 3-*O*-sulfated galactose was present in *N*-glycans of multiple glycoproteins from both PTC and control specimens, as recognized by the O6 mAb and as confirmed by MS analyses. These results provide new insights into the *N*-glycans present in glycoproteins of thyroid cancer and context for further studies of these altered glycans as biomarkers and targets for therapeutics.

## 1. Introduction

Aberrant glycosylation in glycoproteins is a hallmark of cancer, caused in part by changes in the expression of glycogenes and disruption of regular cellular events during cancer progression [1,2,3,4,5]. Fundamentally, glycosylation is the most abundant and varied post-translational modification of proteins [6] and has key roles in organisms at every stage of their development [6,7]. Changes in glycosylation in tumors can occur in many types of glycans, including those linked to Asn and Ser/Thr residues in proteins, e.g., *N*-glycosylation and O-glycosylation, respectively. Such changes include sialylation, fucosylation, branching, elongation and O-glycan truncation, which have been frequently reported to be highly correlative with cancer development [1,8,9,10,11]. Such changes in glycosylation as regulated by glycosyltransferases confer advantages in growth and immunomodulation for cancer cells [12].

A common endocrine malignancy is thyroid cancer, which is among the most common malignancies in adolescents and young adults [13]. Thyroid carcinomas, which have a three-fold higher incidence rate in women than men, are often discovered by physical exam or incidentally on imaging studies [14,15,16]. Papillary thyroid cancers (PTC) account for approximately 80 percent of all thyroid cancer cases [17]. While the overall incidence rate and mortality of thyroid cancer is lower than some other cancers, there has been a steady increase in the PTC incidence rate in recent decades [18]. Furthermore, the mechanisms of thyroid cancer progression and increase in the incidence rate are still unclear [19].

Studies of glycosylation and its functionality related to several thyroid glycoproteins have been undertaken for decades, and the *N*-glycosylation of these glycoproteins includes sialylation, fucosylation and sulfation [10,20,21,22]. Changes in glycosylation can affect the biosynthesis of thyroid hormones and metabolism [23,24]. There is growing information suggesting changes in protein glycosylation, but there is still limited knowledge about protein glycosylation related to PTC [16,25,26,27].

Here, we conducted a comprehensive sulfoglycomic analysis by mass spectrometry and other approaches on surgical specimens of PTC and its nodule controls to establish the baseline for alterations of neutral, sialylated, and sulfated *N*-glycans of PTC in cancer progression. These results show changes in *N*-glycosylation in PTC cancer malignancies and include the determination of the linkage of sulfation on the characterized *N*-glycans. These findings have implications for a better understanding of the fundamental mechanism of malignancy and further progression of PTC cancer as well as indicating potential glycan diagnostic targets for diagnostics and monitoring cancer progression.

## 2. Materials and Methods

### 2.1. Sample Preparation

#### 2.1.1. Tissue Collection

Six paired human tissue samples were collected at the time of standard-of-care thyroidectomies (Table 1). Samples were taken from the pathologically confirmed area of PTC as well as adjacent normal thyroid tissue. Samples were stored at −80 °C until further use. Tissue collection and storage as well as later experimental use was performed following an approved protocol by the Institutional Review Board of Beth Israel Deaconess Medical Center (Protocol #2018P000614, approved 11 October 2018).

#### 2.1.2. Tissue Lysis

Equal wet weights (50 µg) of each PTC and normal thyroid tissue samples were minced in MEPBS (β-mercaptoethanol in phosphate-buffered saline) with protease inhibitors (Roche, Basel, Switzerland) into small pieces, followed by further homogenization in 500 µL ice-cold lysis buffer (50 mM TRIS, 150 NaCl, 1.0% *w*/*v* Triton-X, pH 7.6) using a Dounce homogenizer. Protein concentration was determined using the Pierce BCA Protein Assay Kit (Thermo Fisher Scientific #23255, Waltham, MA, USA).

### 2.2. Lectin Blotting

Lectin blotting was performed as previously described [29]. Thyroid lysates from the PTC and normal paired samples were digested by PNGase F, neuraminidase A (neuA), neuraminidase S (neuS), and O-glycosidase (New England Biolabs, Inc., Ipswich, MA, USA) at 37 °C for 16 h. Lysates were prepared with 4X Laemmli protein sample buffer (Bio-Rad, #1610747, Hercules, CA, USA) with 10% β-mercaptoethanol (*v*/*v*), and denatured for 5 min at 95 °C. A total of 30 µg of protein was loaded per well (GenScript 4–20% Expressplus PAGE 12-lane Gels, Piscataway, NJ, USA). Gels were run using the MiniProtean Tetra Vertical Electrophoresis system (Bio-Rad, #1658004, Hercules, CA, USA) at 140 mV for one hour. Proteins were stained with Coomassie Brilliant Blue (CBB) or transferred to a nitrocellulose membrane using the Trans-Blot Turbo Transfer System (Bio-Rad, #1704150, Hercules, CA, USA). Membranes were incubated with 5% (*w*/*v*) bovine serum albumin (BSA) in TRIS-buffer saline with 0.05% Tween 20 (TBST) for 1 h at room temperature followed by incubation with biotinylated lectins concanavalin A (ConA), *Sambucus nigra* agglutinin (SNA), *Maackia amurensis* lectin (MAL-I), *Phaseolus vulgaris* leucoagglutinin (PHA-L), *Phaseolus vulgaris* erythroagglutinin (PHA-E) (Vector Labs, Newark, CA, USA) and the 06 antibody (lamprey-derived recombinant anti-3-0-sulfo-Gal) [30]. Horseradish peroxidase (HRP)-labelled goat anti-mouse IgG or streptavidin secondary antibodies were used at 1:5000 dilution in TBST, using SuperSignal West Pico Chemiluminescent Substrate (Thermo Fisher Scientific, Waltham, MA, USA). Only 5 of the 6 samples had enough material for the blotting experiments.

### 2.3. N-Glycan Extraction and Permethylation

Glycoproteins within the thyroid cell lysates were processed chemically and enzymatically to release the *N*-glycans as described previously [31] (Supplementary Figure S1). Briefly, 200 µg of proteins were chemically reduced (inter and intra di-sulfide bonds) and carboxymethylated by sequential treatments with dithiothreitol and iodoacetamide. Trypsin was applied at a 1:50 (*w*/*w*) ratio for overnight proteome digestion at 37 °C. *N*-glycans were released from glycopeptides by 24 h treatment with PNGase F (New England Biolabs, Inc., Ipswich, MA, USA) and separated from remaining peptides by C18 Sep-Pak reversed phase column (Waters Corp, Milford, MA, USA).

Released *N*-glycans were permethylated by the sulfoglycomics method as described previously with minor amendments [32]. In brief, *N*-glycans were permethylated in 1 mL sodium hydroxide-DMSO slurry with 500 µL iodomethane at 4 °C for 3 h with gentle vortex. Permethylated glycans were cleaned and separated to neutral (potentially sialylated but non-sulfated) and charged fractions (potentially sulfated) by mixed anion exchange (MAX) column (Waters Corp, Milford, MA, USA). Collected glycan fractions were further cleaned by C18 column chromatography (Waters Corp, Milford, MA, USA) prior to mass spectrometry analysis.

### 2.4. Neuraminidase Treatments

Glycans were purified after PNGase F treatment to release *N*-glycans and desialylated by treatment with sialidase S (from *Streptococcus pneumoniae*, New England Biolabs, Inc., Ipswich, MA, USA) and sialidase A (from *Arthrobacter ureafaciens*, New England Biolabs, Inc., Ipswich, MA, USA) at 37 °C for 24 h. The treated glycans were further purified by C18 column for permethylation and mass spectrometry analysis.

### 2.5. MALDI-TOF MS Analysis

Permethylated glycans were dissolved in 10 µL methanol. An aliquot of each sample (1 µL) was then mixed with 1 µL of matrix (20 mg/mL of 2,5-dihydrobenzoic acid in 50% (*v*/*v*) aqueous methanol) and spotted onto an MTP 384 polished steel BC target plate (Bruker Daltonics, Billerica, MA, USA). MS data were acquired on a Bruker UltraFlex II Matrix-Assisted Laser Desorption/Ionization-Time of Flight Mass Spectrometer (Bruker Daltonics, Billerica, MA, USA). The reflector mode was used. Spectra were recorded between 1000–6000 *m*/*z* for *N*-glycans. A signal/noise ratio of at least 2 was considered and only MS signals matching an *N*-glycan composition were considered for further analysis. MS data were exported from flexAnalysis (version 3.4, build 76, Bruker Daltonics, Billerica, MA, USA) for further analysis by mMass [33]. Each mass spectrum was analyzed with peaks assigned and annotated based on the Consortium for Functional Glycomics glycan database.

### 2.6. Linkage Analysis for Sulfated N-Glycans

Linkage analysis was conducted on permethylated *N*-glycans as described previously [34]. In brief, permethylated *N*-glycans were hydrolysed in 2 M TFA at 120 °C. Monosaccharides released from the hydrolysis were reduced in 2 M ammonia solution with sodium borodeuteride and modified by acetic anhydride at 100 °C. The resulting partially methylated alditol acetates (PMAA) were cleaned by water/chloroform partitioning and redissolved in hexane for GC-MS linkage analysis.

### 2.7. Analysis of RNA Sequencing Data

RNA sequencing data for twenty additional paired PTC and normal human thyroid tissue samples were acquired from The Cancer Genome Atlas (https://portal.gdc.cancer.gov/, accessed on 28 October 2022). Glycogene expression was compared between normal and PTC samples in units of fragments per kilobase of exon per million mapped fragments (RPKM).

### 2.8. Statistical Analysis

For glycomic analysis, individual and groups of glycans were compared using unpaired, two-sample *t*-tests. Analysis of RNA sequencing data was performed using a paired, two-sample *t*-test. For all statistical tests, *p* values < 0.05 were considered statistically significant. All statistical analyses were performed using Stata version 16.1 (StataCorp LLC, College Station, TX, USA).

## 3. Results

### 3.1. Western Blot Reveals Patterns Unique Aspects of N-Glycosylation and Presence of 3-0 Sulfated Galactose Residues in Normal Thyroid Tissues

**Figure 1:**
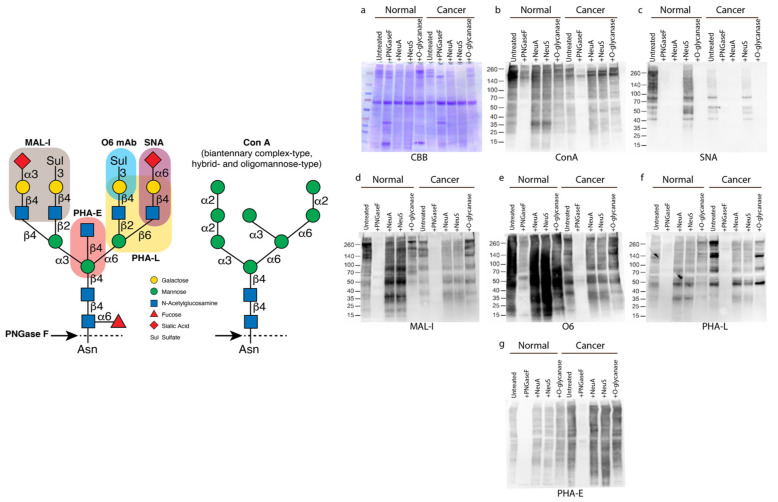
Lectin blot of normal human thyroid tissue and papillary thyroid cancer (PTC). Normal and PTC extracts of a single patient sample examined after reducing SDS-PAGE and transfer for interactions by Western blot techniques with a variety of plant lectins and the O6 mAb, whose glycan specificities are indicated in the graphic at left. Prior to gel electrophoresis, the samples were treated with or without PNGaseF (see graphic), neuraminidase A (NeuA) or neuraminidase S (NeuS) (remove sialic acid), or O-glycanase (removes core 1 O-glycans), and proteins resolved on SDS-PAGE. Gels stained with either Coomassie Brilliant Blue solution (CBB, **a**) or analyzed by Western/lectin blot probed with ConA (**b**), SNA (**c**), MAL-I (**d**), O6 VLR antibody (**e**), PHA-L (**f**), or PHA-E (**g**). (**a**) serves as a loading control for (**b**–**g**). This set of gels is a representative set from 1 patient sample of 5 patients (one sample did not have enough material remaining after processing to perform these assays). Original western blots can be found at Supplementary Materials.

We analyzed a total of five paired human thyroid specimens freshly obtained through thyroidectomies and represented confirmed area of PTC as well as adjacent normal (benign) thyroid tissue. We prepared tissue homogenates, which were then evaluated for specific glycan epitopes using lectins and antibodies in Western blot-type approaches [35,36]. The specificities of these reagents are indicated in the graphic in Figure 1. With a focus on normal specimens first, we observed that ConA, a lectin that binds oligomannose, hybrid, and many biantennary complex-type *N*-glycans [29,37] (Figure 1), bound to multiple glycoproteins indicating the broad expression of such *N*-glycans (Figure 1a,b). Enzymatic treatment with PNGase F, which removes all *N*-glycans [38], exhibited efficient removal of N-glycans resulting in significantly reduced ConA staining. Staining by SNA, a lectin that binds α2-6-linked sialic acids [39], revealed the common presence of α2,6-sialylation on *N*-glycans of many glycoproteins (Figure 1c). This staining was prominent in the normal tissue, and was eliminated by neuraminidase A, which releases α2-3, α2-6, α2-8,and α2-9 sialic acid residues [40], as well as by treatment PNGase F; however, staining by SNA was unaffected by neuraminidase S which hydrolyzes only α-2,3-linked sialic acids [40]. Treatment with O-glycanase (removes core 1 O-glycans) confirms that the subsequent binding of each respective lectin is to N-glycans. These results indicate that a large number of glycoproteins in normal thyroid express *N*-glycans with α2,6-sialylation.

Staining with MAL-I, a lectin that binds glycans expressing either α2-3-linked sialic acids [41] or 3-*O*-sulfated galactose residues [42], indicated the presence of such determinants in multiple glycoproteins, which was strongly reduced by PNGase F, indicating their expression on *N*-glycans. However, MAL-I binding, unlike that of SNA, was not eliminated by treatments with either neuraminidase A or S, suggesting that it is bound to the 3-*O*-sulfate galactose residues on *N*-glycans (Figure 1d). It also appeared that binding was increased somewhat by desialylation, which might expose more binding sites to the lectin. To address the potential presence of 3-*O*-sulfated galactose on *N*-glycans as being responsible for much of the MAL-I binding, we observed intense staining of multiple glycoproteins by O6, a monoclonal antibody (mAb) that specifically binds glycans with 3-*O*-sulfated galactose residues [30]. Staining occurred toward *N*-glycans, as binding was largely diminished by treatment with PNGase F (Figure 1e). These results indicate that normal thyroid tissues express many glycoproteins whose *N*-glycans display α2-6 sialylation along with 3-O-sulfated galactose residues, and there is relatively low expression of α2-3 sialylated *N*-glycans.

### 3.2. Papillary Thyroid Cancer Tumors Are Characterized by Complex-Type, Branched N-Glycans

We observed that in comparison to paired normal thyroid tissue, the glycoproteins in matched PTC tumor specimens demonstrated a reduction in staining by ConA, SNA, and MAL-I (Figure 1b–d). Additionally, binding by O6 antibody appeared somewhat reduced in PTC samples (Figure 1e). In contrast, binding by PHA-L, a lectin that binds *N*-glycans expressing β1-2- and β1-6 branches [43], was generally increased in PTC specimens, suggesting an increase in *N*-glycan branching; binding of PHA-L was sensitive to PNGase F treatment, as expected (Figure 1f). Similarly, staining by PHA-E, which binds to bi-, tri-, tetra-antennary *N*-glycans containing the β1,4-GlcNAc bisected residue [43], was increased in PTC samples relative to normal thyroid tissue samples (Figure 1g).

**Figure 2:**
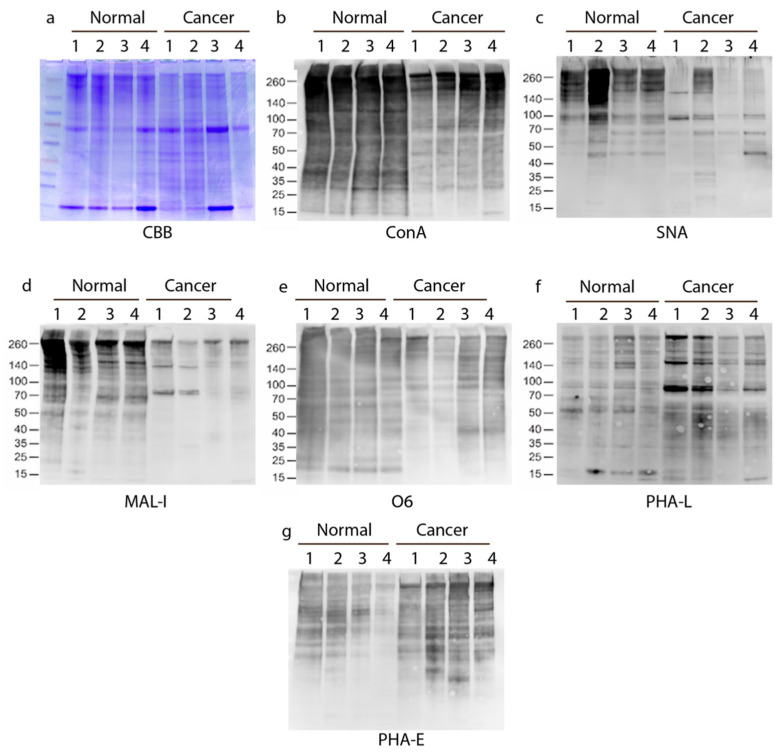
Lectin blot cross-comparison of four paired normal and cancerous human thyroid tissue homogenates. Tissue extracts from four of six specimens for normal and PTC were resolved on SDS-PAGE and stained with CBB (**a**), as in Figure 1, and in analyzed by Western/lectin blot using ConA (**b**), SNA (**c**), MAL-I (**d**), O6 VLR antibody (**e**), PHA-L (**f**), or PHA-E (**g**). (**a**) serves as a loading control for (**b**–**g**). Original western blots can be found at Supplementary Materials.

The differences between normal and PTC seen in Figure 1a–g were recapitulated in the set of four additional samples tested with the same panel of lectins and antibodies in Figure 2a–g. While there are obvious variations seen between each sample within a set, as would be expected in human samples, there are clear trends in terms of higher binding to normal tissue with ConA, SNA, MAL-I (Figure 2b–d), and somewhat with O6 (Figure 2e), while the PTC tissues showed overall more intense binding by PHA-L and PHA-E (Figure 2f–g).

### 3.3. Differential Expression of Glycogenes in PTC Versus Normal Specimens

**Figure 3:**
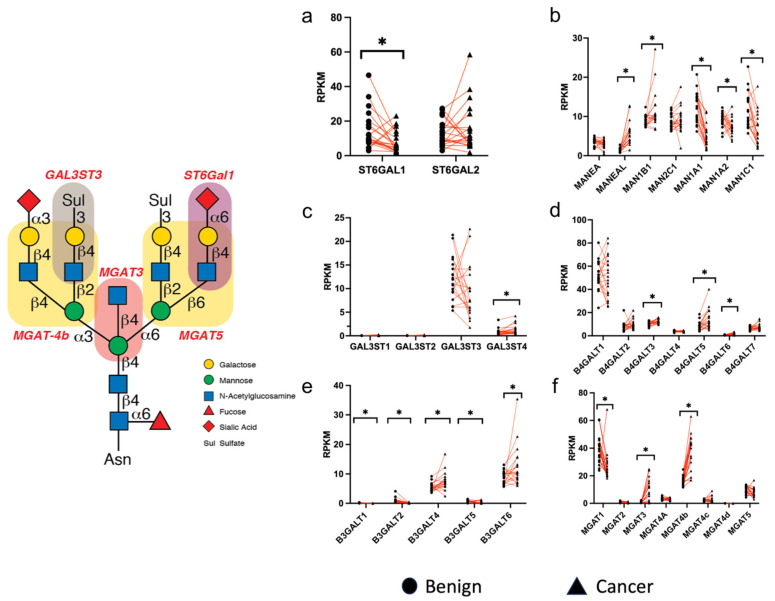
Differential expression analysis of glycogenes. RNA sequencing data from The Cancer Genome Atlas were compared between paired normal (benign) and PTC (cancer) individual samples in units of fragments per kilobase of exon per million mapped fragments (RPKM). Graphic at left depicts the products of some of the glyco-enzyme activities. Panels are grouped by different types of glyco-enzymes, including (**a**) sialyltransferases, (**b**) mannosidases, (**c**) sulfotransferases, (**d**) β1-4galactosyltransferases, (**e**) β1-3galactosyltransfeases, and (**f**) *N*-acetylglucosaminyltransferases. * = *p* < 0.05 when comparing levels in benign and cancer tissues. For all panels, n = 20 for each specified benign and cancer group.

Because of variables in the specimens at the time of collection, we could not ensure the integrity of their RNA preparations. Therefore, we used RNAseq data from The Cancer Genome Atlas to compare the relative expression of selected glycogenes involved in biosynthesis of *N*-glycans, and their sialylation and sulfation between paired tissue samples (Figure 3). These data are more reliable as care is taken to ensure preservation of RNA in the samples, as well as providing an independently valid set of tissue samples for comparison.

Evaluation of the expression of *ST6GAL1*, which encodes the primary enzyme that modifies *N*-glycans with α2-6-linked sialic acid [44,45,46,47], revealed higher expression in normal thyroid tissues compared to PTCs, whereas there was no significant difference in expression of ST6Gal2, which may not efficiently modify *N*-glycans [48] (Figure 3a). Expression of mannosidase genes *MANEAL* and *MAN1B1* were significantly increased in PTC samples, while *MAN1A1*, *MAN1A2*, and *MAN1C1* all demonstrated significant reductions in expression in PTCs relative to normal samples (Figure 3b). The expression of *GAL3ST3*, a potential 3-*O*-sulfotransferase that modifies the Gal residues in LacNAc sequence (Galβ1-4GlcNAc-R) in glycoproteins with 3-*O*-sulfate [49], exhibited relatively high, but variable, expression in the normal and PTC samples; by contrast, *GAL3ST4*, a 3-*O*-sulfotransferase that acts exclusively on galactose in the *O*-glycan Galβ1-3GalNAca1-Ser/Thr in glycoproteins, exhibited low expression but appeared to be statistically elevated in PTC (Figure 3c). In the *B4GALT* family, which encode enzymes that add β1-4-linked galactose to glycans, three of them, T3, T5, and T6 showed statistically significant increases in PTC compared to normal (Figure 3d). All five of the tested *B3GALT* genes, which encode enzymes that add β1-3-linked galactose to glycans, showed significant changes between normal and PTC (Figure 3e). There was varied expression of *MGAT* glycogenes, with a notable increase in PTC specimens in the expression of *MGAT3*, which generates bisected *N*-glycans with the β1-4GlcNAc residue linked to mannose (Figure 3f) [50], and *MGAT4b* [51,52], which branches *N*-glycans (Figure 3f). These results are generally consistent with observations on *N*-glycan expression using the approaches in Figure 1 and Figure 2, including changes in enhanced bisection, enhanced branching, and decreased sialylation, in PTC samples compared to normal. In addition, the results strongly indicate that *GAL3ST3* is significantly expressed in all normal and PTC samples, consistent with observed presence of 3-O-sulfated galactose in *N*-glycans.

### 3.4. N-Glycomics Analysis

The *N*-glycans of normal tissue and PTC samples were more directly characterized by mass spectrometry (MS)-based glycomics analyses to confirm the findings from Western blotting. The *N*-glycans were released by PNGase F treatment of lysates of thyroid specimens. The *N*-glycans of a benign (normal) thyroid and PTC homogenates are shown in different data panels as they were fractionated to neutral and low charged (sialylated) and more anionic species (primarily sulfated) by MAX columns after permethylation. This permitted us to simplify the mass spectrometry analysis, and facilitated the ionization of low abundant charged species, without suppression from the more abundant neutral glycans.

**Figure 4:**
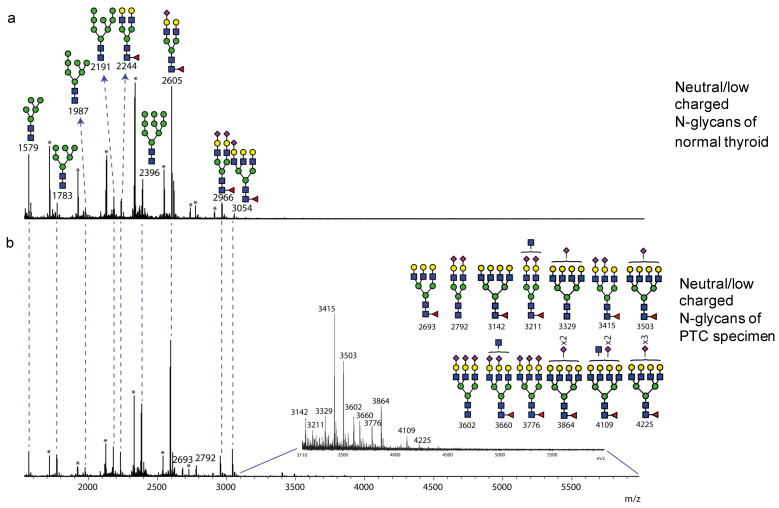
Neutral *N*-glycan profile of normal thyroid tissue and PTC from matching patient. Neutral *N*-glycans released from thyroid homogenates by PNGase F following mild permethylation and fractionation by MAX columns. The data were acquired by MALDI-TOF MS in the positive ion mode. The medial mass-to-charge range is shown from *m*/*z* 1550 to 6000. The ions corresponding to fully permethylated glycan structures are labelled with its molecular mass, putative glycan structures are also assigned to each labelled mass. Signals with identical molecular mass are matched by dash lines between the spectra. Signals not corresponding to fully permethylated *N*-glycan structures are noted with an asterisk *. (**a**) Neutral *N*-glycan profile of thyroid normal tissue; (**b**) Neutral *N*-glycan profile of PTC.

The neutral and low charged *N*-glycan fractions of thyroid benign tissue (Figure 4a) from the matching patient, whose Western blotting results are shown in Figure 2, are dominated by two bi-antennary complex-type *N*-glycan structures (*m*/*z* 2605 and 2966), and abundant presence of oligomannose *N*-glycans (*m*/*z* 1579, 1783, 1987, 2192 and 2396). The complex-type *N*-glycans are mainly core-fucosylated with various degrees of terminal sialylation; tri-antennary structures (such as *m*/*z* 3054) are comparatively minor, and even fewer tetra-antennary structures are present, but in relatively lower amounts. In the PTC, except for a similar presence of high mannose and bi-antennary structures, more branched tri- and tetra-antennary structures are identified as well as an increase in signal abundance (e.g., *m*/*z* 3054, 3415 and 3503); an additional hexNAc residue, presumed to be GlcNAc, occurs within some complex structures (*m*/*z* 3211, 3660 and 4109), consistent with the presence of bisecting GlcNAc (Figure 4b). The changes in neutral *N*-glycans from normal tissue to PTC in branching and GlcNAc bisection are consistent with the Western blot staining and binding by PHA-E (Figure 1 and Figure 2). Variation between samples from patient to patient is expected, whereas the observed changes in *N*-glycosylation are generally consistent in all tested paired samples (n = 5) from normal tissues to PTC.

### 3.5. N-Sulfoglycomics Analysis

**Figure 5:**
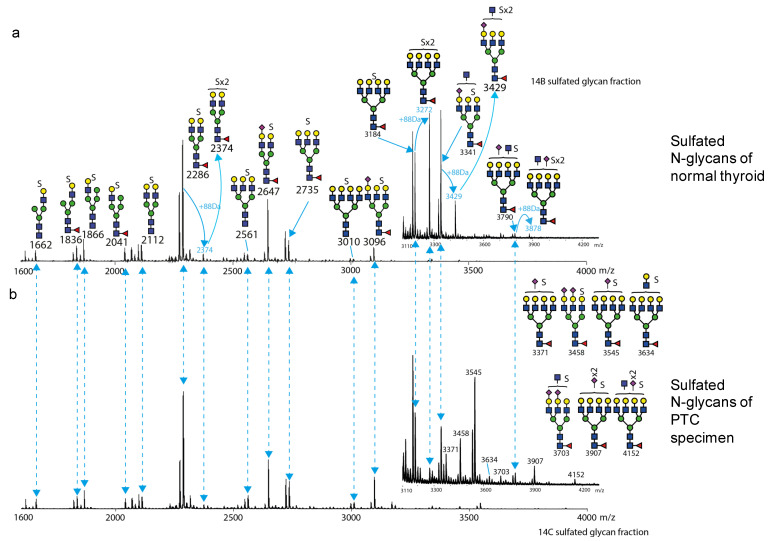
Sulfo *N*-glycan profile of normal thyroid tissue and PTC from matching patient. Sulfated *N*-glycans from the charged glycan fraction were PNGase F released, permethylated, and fractionated by MAX column. The MS profile spectra were acquired in the negative ion mode MALDI-TOF MS. The medial range is *m*/*z* 1600 to *m*/*z* 4000 as shown. The inset medial range shown is from *m*/*z* 3110 to 4200. The fully permethylated mono-sulfated *N*-glycans are labelled with black number and their putative structures are also assigned with a “S” symbol above the cartoon to represent the presence of a sulfo group. Ions corresponding to putatively di-sulfated *N*-glycans are labelled with blue numbers and the +88Da mass adduct is also labelled. Signals with identical molecular mass for glycan structures are matched between spectra with dash lines. (**a**) Sulfo *N*-glycan profile of normal thyroid tissue; (**b**) Sulfo *N*-glycan profile of PTC.

We found the charged *N*-glycan fractions obtained by chromatography on the MAX column, contain sulfated *N*-glycans. These fractions were pooled and detected in the negative ion mode by MALDI-TOF-MS, the preferred method for retaining sulfated structures [53]. All ions were detected in a singly charged form as [M-H+(n−1)Na]^−^ (n = the number of sulfo groups) [53]. Charged hybrid and/or complex structures were detected in all samples, but no phosphorylated oligomannose *N*-glycans were detected. The sulfo-*N*-glycan pool of normal thyroid tissue is dominated by bi- and tri-antennary complex structures (*m*/*z* 2112, 2286, 2647, 2735 and 3096) with single sulfation (Figure 5a); core-fucosylation and terminal sialylation are also observed on these structures. Abundant corresponding under-permethylation signals (−14 Da) are observed with the assigned sulfo-glycan ions, which may be due to the loss of a sulfo group during permethylation procedure or during ionization, as described previously by Khoo and Yu [53]. This may also be an indirect indication of the presence of di-sulfated structures. Mono-antennary hybrid or complex-type structures (*m*/*z* 1662, 1836, 1866, and 2041) were detected with core-fucosylation. Tetra-antennary *N*-glycans (*m*/*z* 3010, 3184, and 3790) were observed in low abundance; core-fucosylation, terminal sialylation and potential GlcNAc bisection can be found on these structures. Di-sulfated *N*-glycans were observed (*m*/*z* 2374, 3272, 3429, and 3878). The abundance of major mono-, bi- and tri-antennary structures are similar in the PTC and normal tissue samples (Figure 5b); however, there is a relative increase in tri- and tetra-antennary *N*-glycans in terms of signal intensity as well as the structures (*m*/*z* 3371, 3458, 3545, 3634, 3703, 3907, and 4152), which are only seen in PTC samples. Sialylation and potential GlcNAc bisection are mainly found on these PTC specific structures. The complex *N*-glycan with LacNAc extension at *m*/*z* 3634 indicates a potential for further branching or antennary extension by LacNAc building blocks. Interestingly, the relative intensity of the signals for glycans with di-sulfation dramatically decrease on the tri- and tetra-antennary structures (*m*/*z* 3272, 3429, and 3878) in the PTC samples.

**Figure 6:**
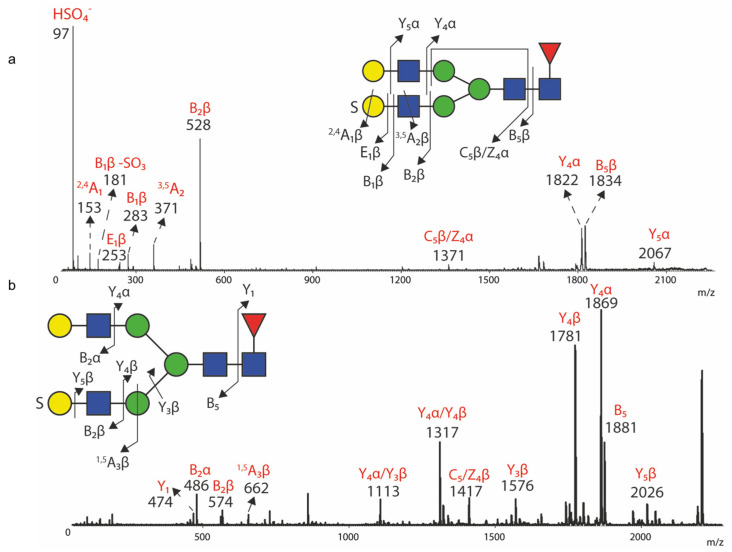
MS/MS fragmentation analyses for *m/z* 2286 and 2332. In-source MS/MS fragmentation analyses were performed by MALDI TOF/TOF for selected ions. Signals for the corresponding fragment ions are labelled with their *m/z* value. The parent ion cartoons are shown with the arrowed lines and numbers representing the corresponding fragments. The symbol “S” is labelled on the glycan cartoon to represent sulfation. The fragment ion assignments are based on the Domon-Costello fragmentation pathways for glycans [56]. (**a**) MS/MS fragment spectrum of *m*/*z* 2286 in the negative mode; (**b**) MS/MS fragment spectrum of *m*/*z* 2332 in the positive mode.

To validate the assigned structural features of sulfated *N*-glycan structures; in-source MALDI tandem mass spectrometry (MS/MS) fragmentation analyses were performed for abundant glycans structures in both the negative ion mode and the positive ion mode. The most abundant bi-antennary structure; *m*/*z* 2286; which contains core fucosylation; was first fragmented in the negative mode (Figure 6a). The sulfate retaining fragment ions were only detectable in the negative ion mode of MALDI-MS/MS. Hence, the observation of diagnostic ion *m*/*z* 97 and B_2_ ion *m*/*z* 528 from the non-reducing end indicates the presence of the sulfo group and sulfo-LacNAc motif; the presence of the B_2_ ion *m*/*z* 283 indicates that the location of the sulfo group is at the terminal galactose residue rather than the internal GlcNAc residue of a sulfo-LacNAc. Moreover; the observation of a series of cross-ring fragmentation ions such as ^2,4^A_1_ ion *m*/*z* 153; E_1_ ion *m*/*z* 253 and *m*/*z* 181 support the presence of the sulfo group on the galactose with a 3-*O* linkage [54]. E ions have been previously described by Khoo and co-workers as unique fragment ions of permethylated glycans when utilizing MALDI-TOF-MS/MS [55]. Core-fucosylation is confirmed by the presence of B_5_ ion *m/z* 1834; additional Y ions at *m*/*z* 2067 (Y_1_) and 1822 (Y_2_) show a sequential fragmentation of the non-sulfo-LacNAc antennae. In addition, fragmentation of the corresponding positive ion, *m*/*z* 2332 ([M+2Na-2H]^+^), (Figure 6b) produces diagnostic ions; including *m*/*z* 474; 486; 574; 1781; 1869 and 1881; to provide complementary information for reconfirming the assigned core-fucosylation and (sulfo)-LacNAc antenna. The presence of Y_5_ ion *m*/*z* 2026 also provides additional information for the presence of terminal sulfo-galactose rather than internal sulfo-GlcNAc.

The mono-sulfated and sialylated bi-antennary structure *m*/*z* 2647 (*m*/*z* 2693 in the positive ion mode) as well as di-sulfated bi-antennary structure *m*/*z* 2374 (*m*/*z* 2420 in the positive ion mode) were both fragmentated (Supplementary Figures S2 and S3). They produced the series of diagnostic ions *m*/*z* 97, 153, 181, 253 and 528 in the negative ion mode, which supports the major sulfo-antenna on these structures as a mono-sulfo LacNAc with 3-*O*-Gal linkage of the sulfo group. The fragmentation of both structures in the positive ion mode not only produced the expected complementary B/Y ions for confirming the assigned *N*-glycan structures, but also provided additional information for the potential presence of minor glycan isomers. Because of the presence of complementary B/Y ions *m*/*z* 935 (B_3_) and *m*/*z* 1781 (Y_4_) in the MS/MS spectrum of *m*/*z* 2693, there may be a minor presence of an isomer with sulfo-sialyl-LacNAc antennae. However, there is no further information that could be extracted from the positive nor negative ion mode MS/MS spectra for the sulfo linkage determination of this motif. In the positive ion mode MS/MS spectrum of *m*/*z* 2420, the presence of B_2_ ion *m*/*z* 486 may indirectly imply the presence of a di-sulfated LacNAc on another antennae. Even with the observation of B_2_ ion *m*/*z* 662, it could indicate a fragment ion from a LacNAc with di-sulfation or a mono-sulfated Gal-GlcNAc-Man arm with a cross-ring fragmentation on the Man residue; there may also be overlap with other minor *N*-glycan structures in the parent ion selection. Therefore, the data are too ambiguous to characterize an additional isomer for *m*/*z* 2374 (*m*/*z* 2420) with di-sulfated LacNAc.

Additionally, *N*-glycans from both normal tissue and PTC were treated by neuraminidases to validate the linkage specificity of salic acids and facilitate the determination of the linkage of observed galactose sulfation. Neuraminidase A, which is non-specific for sialic acid linkage types, removed the capping sialic acid residues, but neuraminidase S, which is specific for α2-3-linked sialic acid could not, indicating the α2-6 linkage specificity on the sialylation of *N*-glycans in thyroid materials (Supplementary Figures S4 and S5). The desialylated *N*-glycans were further permethylated and modified for GC-MS analysis. The GC-MS analysis validated the 3-*O* linkage of sulfo groups on the *N*-glycans (Supplementary Figure S6). These results confirm that many *N*-glycans of thyroid specimens analyzed contain 3-O-sulfated galactose residues, typically 1 per glycan, which is consistent with the strong evidence of the presence of the antigenic epitope in *N*-glycans recognized by the O6 mAb.

## 4. Discussion

This study provides detailed glycomic analyses of *N*-glycans in glycoproteins within PTC specimens and paired normal thyroid tissue. The results demonstrate that there are variations in sialylation, bisection, and sulfation of the *N*-glycans in PTC samples compared to normal specimens. In addition, we identified the presence of 3-*O*-sulfation of *N*-glycans in multiple glycoproteins of all samples, which appeared to be at a lower expression in PTC specimens. The neutral and low-charged (sialylated) *N*-glycan fraction shows a consistent shift of *N*-glycosylation in PTC as branching, reduced sialylation, and increased bisection between patients. The increase in branched *N*-glycans was cross-validated by MS, lectin staining, and RNAseq.

Overall, the results of our study are supported by other recent studies on aspects of PTC protein glycosylation. For example, a recent MS *N*-glycomics study of PTC [26], observed changes in sialyation and branching of *N*-glycans, and another recent study observed an increased in bisected and branched N-glycans [57]. We observed a reduction in sialylation of *N*-glycans in PTC specimens, which is consistent with earlier studies of thyroid lesions [25], but not consistent with a recent *N*-glycomics study of PTC, which suggested an increase in sialylation [26]. Such differences may be due in part to sample preparation, as we used fresh tissue and completely solubilized glycoproteins for analysis, whereas other analyses used formalin-fixed paraffin-embedded tissues [26], which may affect the enzymatic release of *N*-glycans. There could also be variations in the diversity among patients. A key aspect of our approach was to analyze the materials by SDS-PAGE and Western blotting with lectins and the O6 mAb to identify the broad distribution of modifications on multiple glycoproteins, the completeness of reactions with PNGase F and neuraminidase treatments. Altogether, our results suggest a degree of variation in glycosylation between patient samples, but in paired materials of the same individual, we observed differences in glycosylation of normal versus PTC specimens.

We observed an increase in GlcNAc bisection of *N*-glycans of PTC specimens in this study, also consistent with significant staining of glycoproteins with the plant lectin PHA-E, which binds this modification [43] (Figure 3). This bisection feature was also reported to be increased in a recent *N*-glycomics study of PTC [26]. The increased level of GlcNAc bisection, which is consistent with elevations in expression of *MGAT3* in PTC specimens as shown here, suggest that future studies with PTC samples should further assess its association with cancer progression status, as other studies have reported that increased bisection of *N*-glycans is associated with progression, metastasis, and poor survival in other cancers [58,59,60].

We also observed elevations in expression of *MGAT4b*. *MGAT4b* [61] along with *MGAT5* [62], are responsible for addition of GlcNAc residues in generating branched *N*-glycans (Figure 3), which include tri- and tetra-antennary species, many of which are recognized by the lectin PHA-L [43,63]. We observed greater staining of PTC specimens by PHL-L compared to normal specimens. Enhanced branching of *N*-glycans is commonly seen in many human tumors and is associated with growth signaling and galectin interactions in the glycocalyx [11,64,65,66,67]. *β3GALT6*, which we observed to be elevated in many PTC specimens, is involved in elongation of the core region of glycosaminoglycans [68], and is involved in enhanced FGF-mediated signaling [69]. We did not examine the structures or biosynthesis of glycosaminoglycans in our materials, but these results suggest a potential involvement in thyroid papillary carcinomas.

We were surprised to find widespread expression of 3-O-sulfated galactose on multiple glycoproteins in normal and PTC samples, as evidenced by staining with the O6 mAb, specific for this modification [30]. Our observations in this regard are relatively unique, as there is little information regarding the expression of this determinant on human cancers. This modification appears to be expressed mainly as mono-sulfated species and found particularly on bi-antennary *N*-glycans (Figure 6). However, our data suggest that di-sulfated and even further multi-sulfated glycans might be present, but further characterization was not possible, primarily due to the general instability of instability of sulfo groups during ionization in MS [53], as well as the instrumental challenges to fragment and sequence the ions for potentially multi-sulfated structures with only trace-level abundance.

The dominance of a monosulfated glycan composition is different from the dominant glycan composition of complex structures in the neutral glycan fractions, which is bi-antennary with mono-sialylation. The changes in the sulfated *N*-glycans in PTC are generally consistent with those observed in the neutral *N*-glycans, which may suggest that sulfation is not selective for specific thyroid *N*-glycans. Interestingly, in the MS profile of sulfated glycan fractions, there was little evidence for glycan masses containing a singly sialylated LacNAc antenna and a single sulfate group (Figure 5). The negative mode MS/MS fragmentation analysis for the sulfated structure with sialylation demonstrates that for the most part sulfation and sialylation are not located on the same antennae (Supplementary Figure S2). The linkage of sulfation on 3-O-position of galactose is consistent with the detailed cross-ring fragmentation of the sulfate-bearing galactose residue in the MS/MS analysis. The dominant sialylation on thyroid *N*-glycans is characterized as α2,6 linkages by MS, neuraminidase treatments, and lectin blot analysis. Thus, there may be little direct competition for the 3-O-position on the galactose residue, as we observed only low levels of α2-3 sialylation of galactose, and thus the *GAL3ST3* enzyme may have enhanced access for addition of 3-O-sulfate to galactose. It was observed previously that enhanced expression of *GAL3ST3* leads to extensive 3-O-sulfation of galactose residues in multiple *N*-glycans and in the case of expression in Chinese hamster ovary, this competes well with α2-3 sialylation of galactose, thus blocking sialylation [42]. Finally, while O-6 mAb staining by Western blotting (Figure 1e and Figure 2e), suggest a potential reduction in levels of 3-O-sulfation in PTC specimens, the expression of *GAL3ST3* does not appear to be significantly altered in normal versus PTC samples (Figure 3c). Nevertheless, there could be other factors that contribute to a decrease in the epitope, such as other types of *N*-glycan modifications and overall Golgi organization and dynamics, which are often disrupted in cancers [70].

In regard to analysis of sulfated glycans, the MS/MS fragmentation for the glycans in the positive mode may provide complimentary BY fragment ions, which may be benefited from the superior ionization capacity under our instrumental setting. This indirectly suggested a minor abundance of mono-sulfation on the sialyl-LacNAc antennae, which we speculate could be 6-*O*-sulfate GlcNAc, as was reported in an earlier study for the carbohydrate composition of thyroglobulin [21]. However, this requires further validation of sulfation and sulfoglycomics in future studies.

The functions of sulfated *N*-glycans are not well understood, but there is growing evidence suggesting that they may be important in multiple pathways [71]. The modification of glycans with 3-O-sulfated galactose is predicted to relatively abundant in only a few specific cells and tissues based on expression of *GAL3ST3* [72]; expression is highest in the thyroid gland, kidney, and in certain neuronal cell types. One of the earliest studies in this regard was the discovery of 3-O-sulfation of galactose residues thyroid glands and especially in thyroglobulin [21,73,74]. While the functions of galactose sulfation in *N*-glycans are unclear, there are proposals that sulfation of glycans serves as a mechanism to regulate or fine-tune glycan interactions with Siglecs, a family of sialic acid-binding proteins expressed mainly in lymphocytes and involved in immune evasion [75]. In addition, there is evidence that galectin-8, a soluble lectin expressed in human thyroid carcinomas and other tumor types [76,77], binds to 3-O-sulfated galactose residues in glycans, which be involved in various pathways of lymphocyte regulation and stimulation [78,79,80]. However, at present, the specific roles of 3-O-sulfated galactose in *N*-glycans of thyroid glycoproteins are unknown and require further study.

In the present study, we found that core fucosylation was common on most complex *N*-glycans in both groups (Figure 4 and Figure 5), and therefore we did not examine expression of the *FUT8*, which encodes the enzyme responsible for its generation. However, a prior study suggested that *FUT8* was overexpressed in thyroid cancer [81], and changes in core fucosylation of glycans were documented. However, in that study, the overexpression of *FUT8* was found in one third of the patients evaluated [82], suggesting potential wide variation in this modification in patients. In regard to fucosylation of residues in outer antennae of *N*-glycans, we did not detect significant fucosylation of the branches, although evidence for this has been reported previously [26,27]. Our fucosylation could arise through action of enzymes involved in formation of Lewis and blood group antigens, as seen in tumor malignancies [83], but could also be influenced by the expression of FUT secretor genes depending on the blood type of each individual [84]. Therefore, a larger cohort study with identified blood type classification is needed to elucidate potential correlations of *N*-glycans fucosylation relative to possible blood group antigens.

Several prior studies on thyroid glycosylation focused mainly on thyroglobulin, which is made by thyroid follicular cells. The *N*-glycosylation and sulfation of thyroglobulin was recently studied at the glycomics and glycoproteomics level [85,86]. In the elegant study by Kayili and Salih, it was reported that *N*-glycans of human thyroglobulin display bisected, core-fucosylated, complex *N*-glycans and sulfate residues and there is site-specific expression of distinct glycan types [86]. Interestingly, the loss of *N*-glycosylation on thyroglobulin can cause the disruption of hormone biosynthesis from the thyroid [23,87]. Thus, the glycosylation of *N*-glycans in human thyroglobulin is consistent with the overall patterns of glycosylation we observed here for the *N*-glycans from total thyroid glycoproteins.

Papillary thyroid cancer, which accounts for a large percentage of thyroid cancers, is distinguished by changes in expression of specific oncogenes that drive tumor growth, which includes *RET* proto-oncogene [88,89]. *RET* encodes a 150 kDa glycoprotein, which is a membrane receptor tyrosine kinase that itself is heavily glycosylated with *N*-glycans, which are required for surface localization of the kinase [90]. While the glycosylation of RET has not been well studied in PTC, it is intriguing to consider the possibility that the changes we have observed in *N*-glycan profiles of PTC might be associated with activity of RET, as glycosylation is known to alter functions of receptor tyrosine kinases in other tumor types [91].

## 5. Conclusions

There is a need for further studies on the roles of glycans and their sulfation in normal thyroid function and potential roles in development and progression of PTC, and the malignant status of thyroid nodules. Our observations about changes in protein glycosylation in PTC could provide valuable pre-surgical diagnostic information and aid in the identification of biomarkers for tumors and new therapeutic targets. Therefore, it will be of interest in future studies to target the aberrant glycosylation of PTC and other thyroid cancer subtypes to understand whether they are important in cancer progression involving protein glycosylation, which could help inform innovative therapies and diagnostic approaches.

## Figures and Tables

**Table 1:** PTC clinical information.

Age	Operation	Pre-Operative Diagnosis	Surgical Pathology	Largest Dimen-sion, cm	AI, LI, ETE, Margins	Lymph Nodes	Stage [28]
40	Lobectomy	PTC	PTC	0.7	+LI	-	I (pT1aNx)
61	Total Thyroidectomy + CND + MRND	PTC	PTC	1.8	+LI	+25/52	II (pT1bN1b)
79	Total Thyroidectomy	PTC	PTC	1.8	+LI, +margins	+1/1	II (pT1bN1a)
55	Lobectomy	PTC	PTC	2.2	-	0/3	I (pT2N0)
40	Total thyroidectomy	PTC	PTC, tall cell variant	2.1	-	3/6	I (pT2N1a)
29	Lobectomy, completion thyroidectomy	PTC	PTC	7	-	-	I (pT3aNx)

Notes: CND = central neck dissection; MRND = modified radical neck dissection; AI = angioinvasion; LI = lymphovascular invasion; ETE = extrathyroidal extension; see [28] for stage descriptions.

## Data Availability

The data presented in this study are openly available in GlycoPOST repository for glycomics data, accession number GPST000495.

## Supplementary Materials

The following supporting information can be downloaded at: https://www.mdpi.com/article/10.3390/biom14121482/s1, Figure S1: Schematic of preparation and sulfoglycomic analysis of normal human thyroid tissue and papillary thyroid cancer (PTC); Figure S2: MS/MS in-source fragmentation analyses for negative ion-mode ion *m*/*z* 2647 (top) and positive ion-mode ion *m*/*z* 2693 (bottom). Positive ion-mode data suggests that there are at least two isomers of the proposed composition: one with the sulfate modification on the sialylated galactose (diagnostic fragments shown in blue), and one with the sulfate modification on the non-sialylated galactose (diagnostic fragments shown in pink); Figure S3: MS/MS in-source fragmentation analyses for negative ion-mode ion *m*/*z* 2374 and positive ion-mode ion *m*/*z* 2420. Positive ion-mode data suggests that there are at least two isomers of the proposed composition: one with a sulfate on both arm galactose’s (diagnostic fragments shown in blue), and one with both sulfate modifications on the same arm, with one on the galactose and one on the N-acetylglucosamine (diagnostic fragments shown in pink); Figure S4: Sulfated N-glycan profile of normal thyroid tissue (A) and PTC (B) after Sialidase A treatment; Figure S5: Sulfated N-glycan profile of normal thyroid tissue (A) and PTC (B) after Sialidase S treatment; Figure S6: GC-MS extracted ion chromograms (XIC) for normal thyroid tissue and PTC with standards. Additional Supplementary Material: Original blots.
