# Supplementary material for: Thyroid Carcinoma Glycoproteins Express Altered N-Glycans with 3-O-Sulfated Galactose Residues
Source: Biomolecules. 2024 Nov 21;14(12):1482. doi: 10.3390/biom14121482 (PMC11727208; doi:10.3390/biom14121482)

Broekhuis et al., Supplementary Information

Supplementary Figure Legends

Supplemental Figure S1. Schematic of preparation and sulfoglycomic analysis of normal human thyroid tissue and papillary thyroid cancer (PTC).

Supplementary Figure S2. MS/MS in-source fragmentation analyses for negative ion-mode ion m/z 2647 (top) and positive ion-mode ion m/z 2693 (bottom). Positive ion-mode data suggests that there are at least two isomers of the proposed composition: one with the sulfate modification on the sialylated galactose (diagnostic fragments shown in blue), and one with the sulfate modification on the non-sialylated galactose (diagnostic fragments shown in pink).

Supplementary Figure S3. MS/MS in-source fragmentation analyses for negative ion-mode ion m/z 2374 and positive ion-mode ion m/z 2420. Positive ion-mode data suggests that there are at least two isomers of the proposed composition: one with a sulfate on both arm galactose's (diagnostic fragments shown in blue), and one with both sulfate modifications on the same arm, with one on the galactose and one on the N-acetylglucosamine (diagnostic fragments shown in pink).

Supplementary Figure S4. Sulfated N-glycan profile of normal thyroid tissue (**A**) and PTC (**B**) after Sialidase A treatment. Blue arrows indicate the same glycan peak between samples.

Supplementary Figure S5. Sulfated N-glycan profile of normal thyroid tissue (**A**) and PTC (**B**) after Sialidase S treatment. Blue arrows indicate the same glycan peak between samples.

Supplementary Figure S6. GC-MS extracted ion chromograms (XIC) for normal thyroid tissue and PTC with standards.

Supplemental Figure S1.

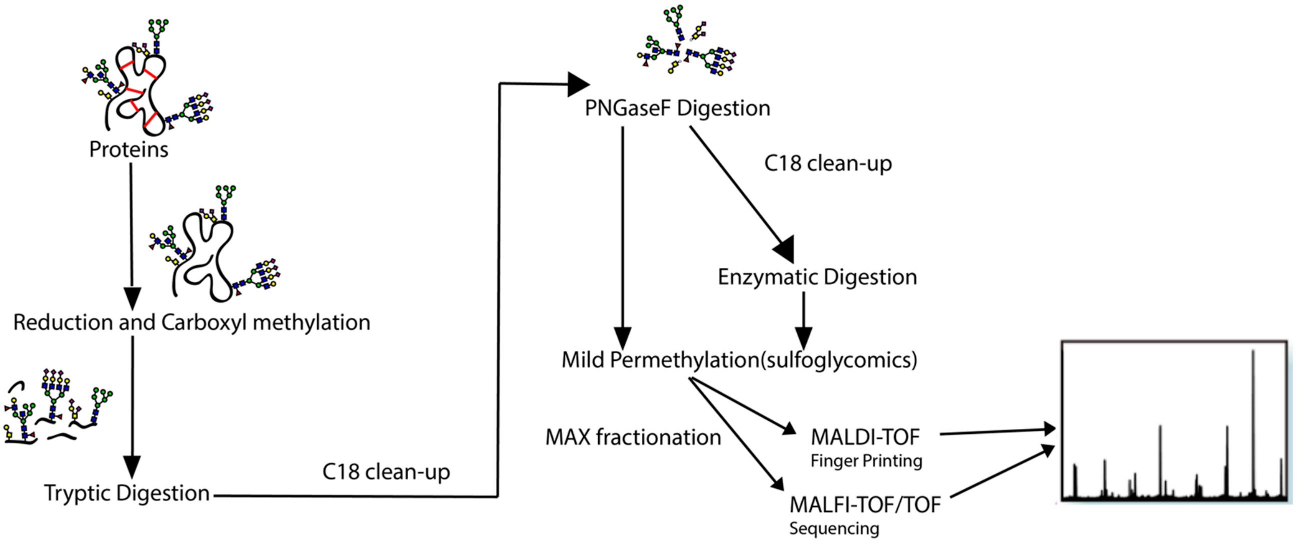

Supplementary Figure S2.

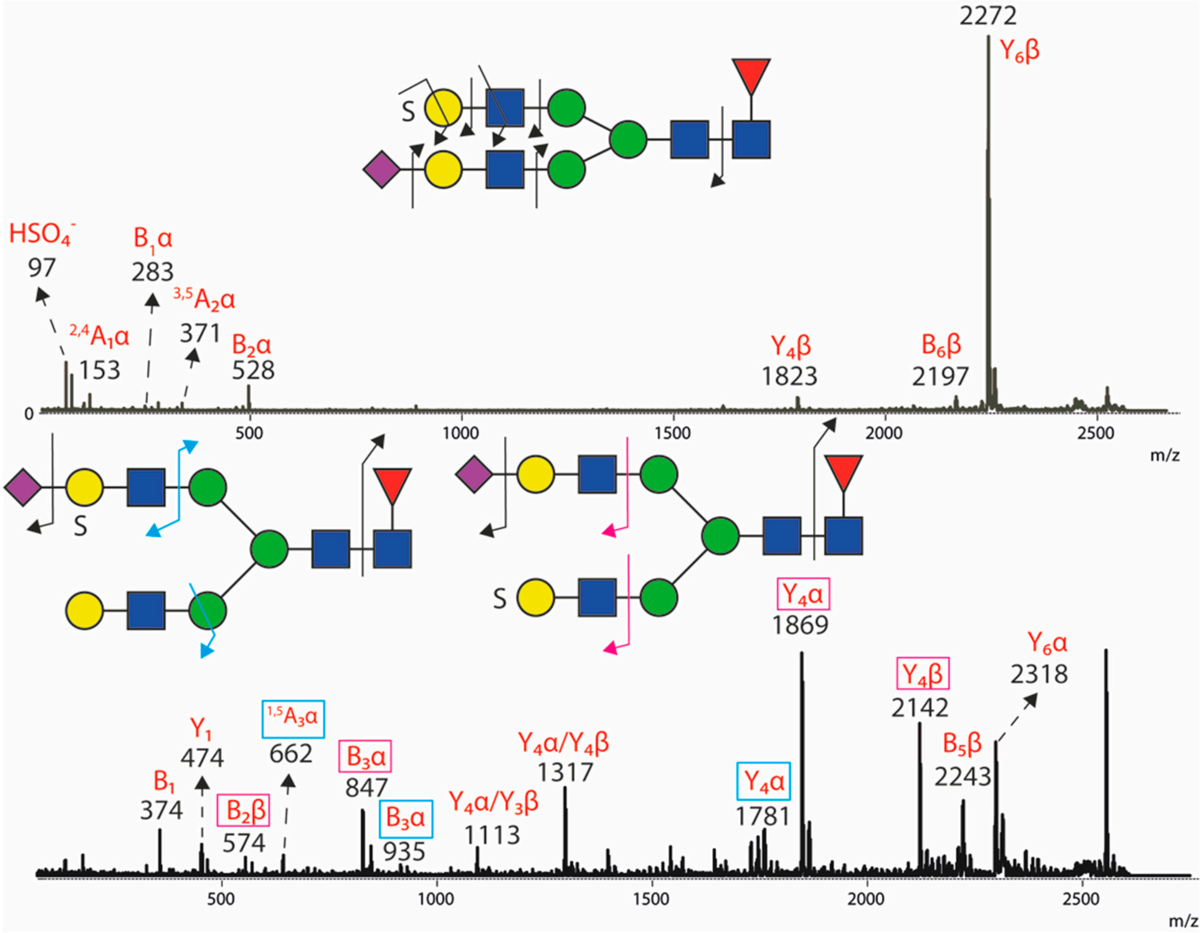

Supplementary Figure S3.

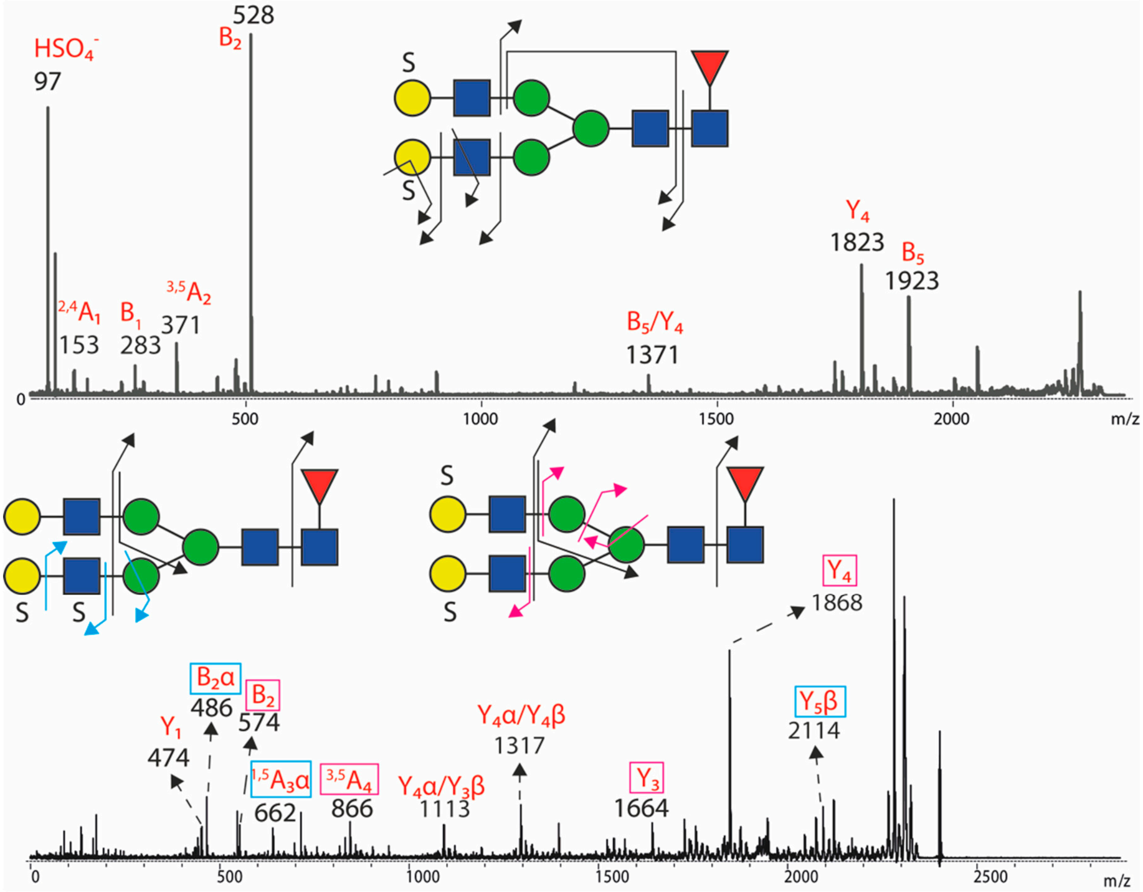

Supplementary Figure S4.

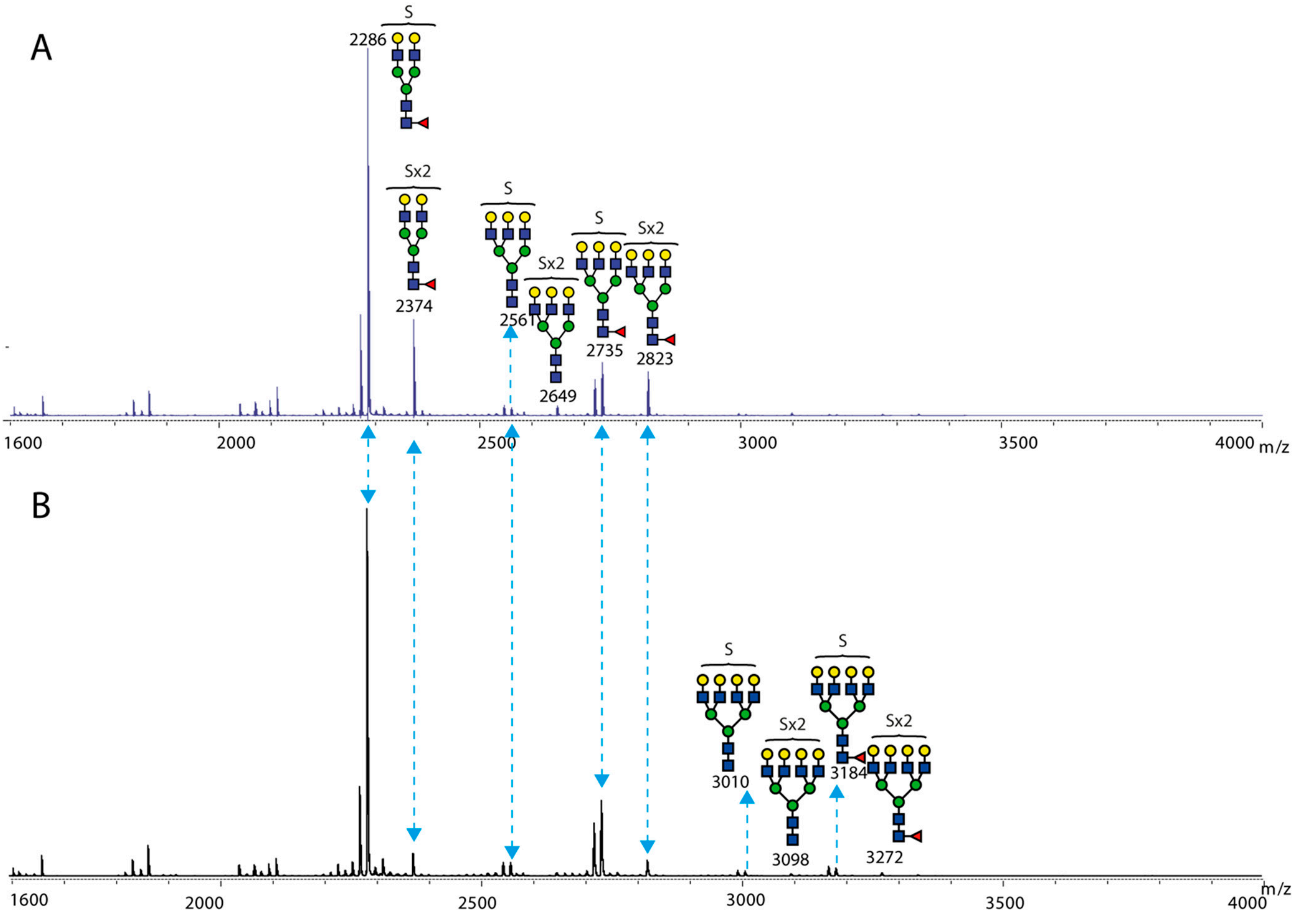

Supplementary Figure S5.

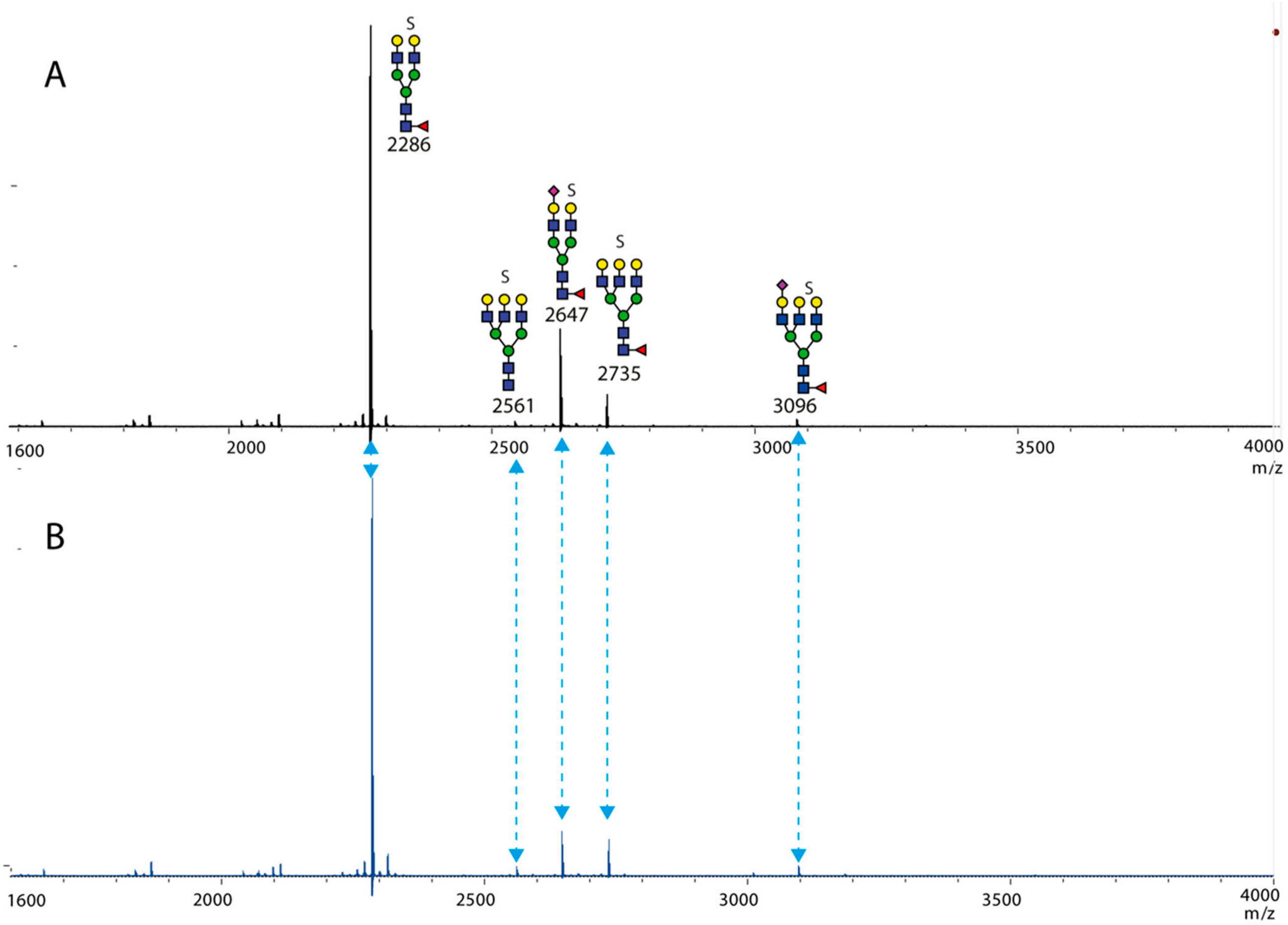

Supplementary Figure S6.

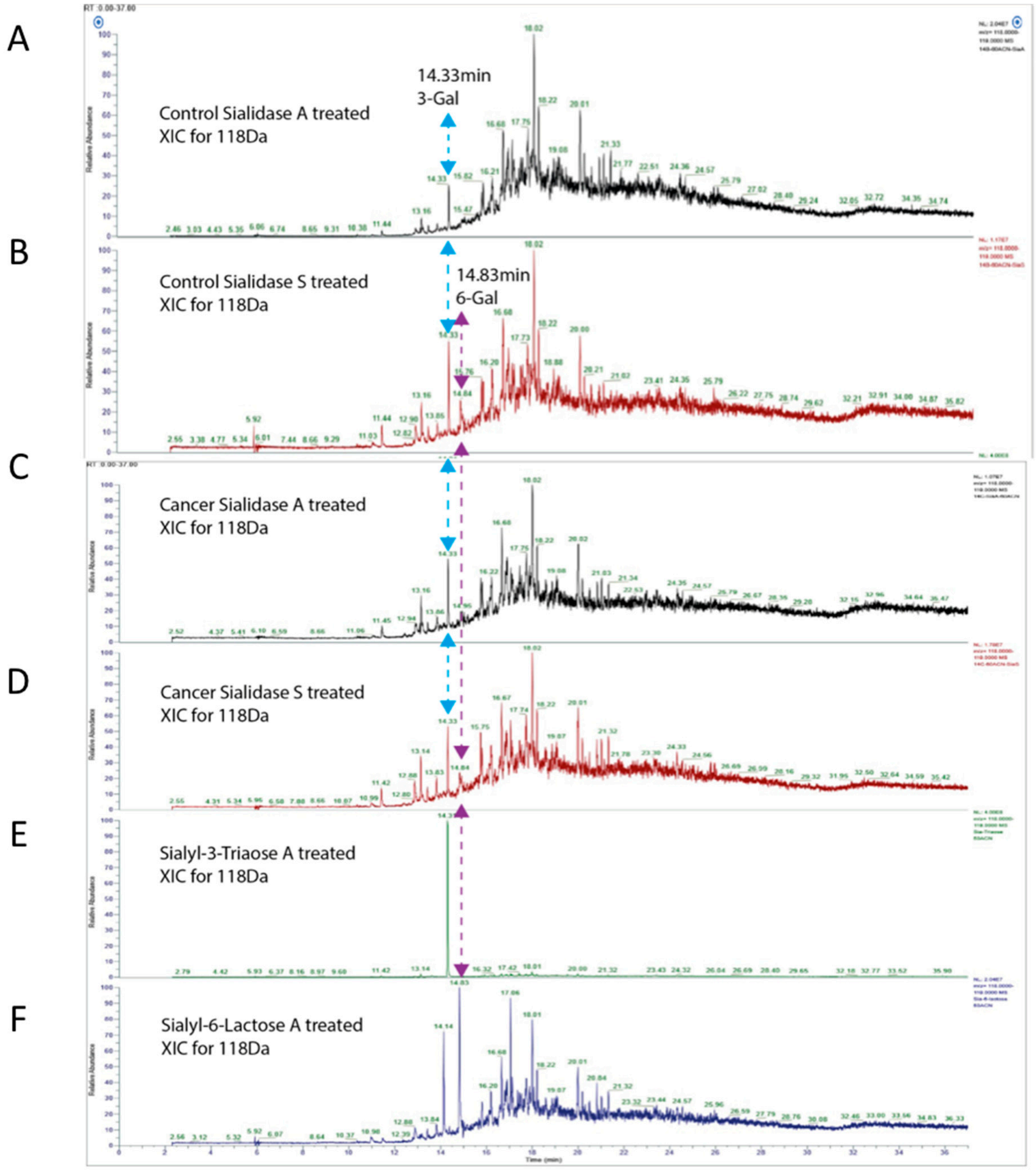

Supplement: Supplementary file 1 [file biomolecules-14-01482-s001.zip › biomolecules-3291742-supplementary figures.pdf]
